# Regional Variation in Tissue Composition and Biomechanical Properties of Postmenopausal Ovine and Human Vagina

**DOI:** 10.1371/journal.pone.0104972

**Published:** 2014-08-22

**Authors:** Daniela Ulrich, Sharon L. Edwards, Vincent Letouzey, Kai Su, Jacinta F. White, Anna Rosamilia, Caroline E. Gargett, Jerome A. Werkmeister

**Affiliations:** 1 The Ritchie Centre, MIMR-PHI Institute of Medical Research, Clayton, Victoria, Australia; 2 CSIRO Manufacturing Flagship, Clayton, Victoria, Australia; 3 Department of Gynecology and Obstetrics, Caremeau University Hospital, Nimes, France; 4 Department of Obstetrics and Gynaecology, Monash Medical Centre, Monash University Melbourne, Victoria, Australia; Massey University, New Zealand

## Abstract

**Objective:**

There are increasing numbers of reports describing human vaginal tissue composition in women with and without pelvic organ prolapse with conflicting results. The aim of this study was to compare ovine and human posterior vaginal tissue in terms of histological and biochemical tissue composition and to assess passive biomechanical properties of ovine vagina to further characterise this animal model for pelvic organ prolapse research.

**Study Design:**

Vaginal tissue was collected from ovariectomised sheep (n = 6) and from postmenopausal women (n = 7) from the proximal, middle and distal thirds. Tissue histology was analyzed using Masson's Trichrome staining; total collagen was quantified by hydroxyproline assays, collagen III/I+III ratios by delayed reduction SDS PAGE, glycosaminoglycans by dimethylmethylene blue assay, and elastic tissue associated proteins (ETAP) by amino acid analysis. Young's modulus, maximum stress/strain, and permanent strain following cyclic loading were determined in ovine vagina.

**Results:**

Both sheep and human vaginal tissue showed comparable tissue composition. Ovine vaginal tissue showed significantly higher total collagen and glycosaminoglycan values (p<0.05) nearest the cervix. No significant differences were found along the length of the human vagina for collagen, GAG or ETAP content. The proximal region was the stiffest (Young's modulus, p<0.05), strongest (maximum stress, p<0.05) compared to distal region, and most elastic (permanent strain).

**Conclusion:**

Sheep tissue composition and mechanical properties showed regional differences along the postmenopausal vaginal wall not apparent in human vagina, although the absolute content of proteins were similar. Knowledge of this baseline variation in the composition and mechanical properties of the vaginal wall will assist future studies using sheep as a model for vaginal surgery.

## Introduction

Pelvic organ prolapse (POP), the herniation of the pelvic organs into the vagina, affects up to 25% of all women [1]. POP predominantly results from vaginal childbirth injury and is exacerbated by ageing, obesity and other factors. However, due to multifactorial reasons the exact aetiology is unclear since young or nulliparous healthy women also develop POP, although at much lower frequency than parous women [2].

The pelvic organs are supported at three different levels by the pelvic floor muscles, the cardinal and uterosacral ligaments and the dense fibromuscular connective tissue of the vaginal wall, termed the endopelvic fascia [3]. The connective tissue of the endopelvic fascia is derived from resident fibroblasts. The main proteins of the extracellular matrix (ECM) are collagen and elastin [4]. The muscularis mainly comprises smooth muscle cells, and along with ECM, is a dynamic structure that undergoes changes in response to the environment. Together collagen type I and III, elastin and smooth muscle cells are mostly responsible for the biomechanical properties of the tissue.

Sheep have been proposed as a suitable model for preclinical studies to perform POP research [5], [6]; sheep have a similar sized pelvic region, large fetal head-maternal pelvis ratio and spontaneously develop POP pre- and postpartum. However to date, no study has compared human and ovine tissue directly to justify sheep as a suitable model for basic scientific research in the field of urogynaecology.

There are an increasing number of reports on the composition of sheep and human vaginal tissue; however, most studies only perform one type of analysis or do not describe the exact location. It is therefore difficult to accurately compare the results and draw conclusions [7], [8]. Regional differences have been observed in the vaginal tissue of rats [9] where the contractility and gross anatomy varied along the vagina. Prolapse repair with synthetic meshes is often associated with severe side effects like pain, infection or erosion. It is necessary to define the tissue at different parts of the vagina to understand prolapse repair. The aim of this study was to assess the variation of histoarchitectural, ECM and biomechanical properties along the length of the vagina of postmenopausal ovine vagina and to compare these findings to human tissue from postmenopausal women.

## Materials and Methods

### ANIMALS

The experimental procedures and sheep husbandry were approved by the Monash Medical Centre Animal Ethics Committee A. Border Leicester Merino sheep were housed in the Monash Animal Service facilities in compliance with the National Health and Medical Research guidelines for the care and use of laboratory animals.

**Figure 1:**
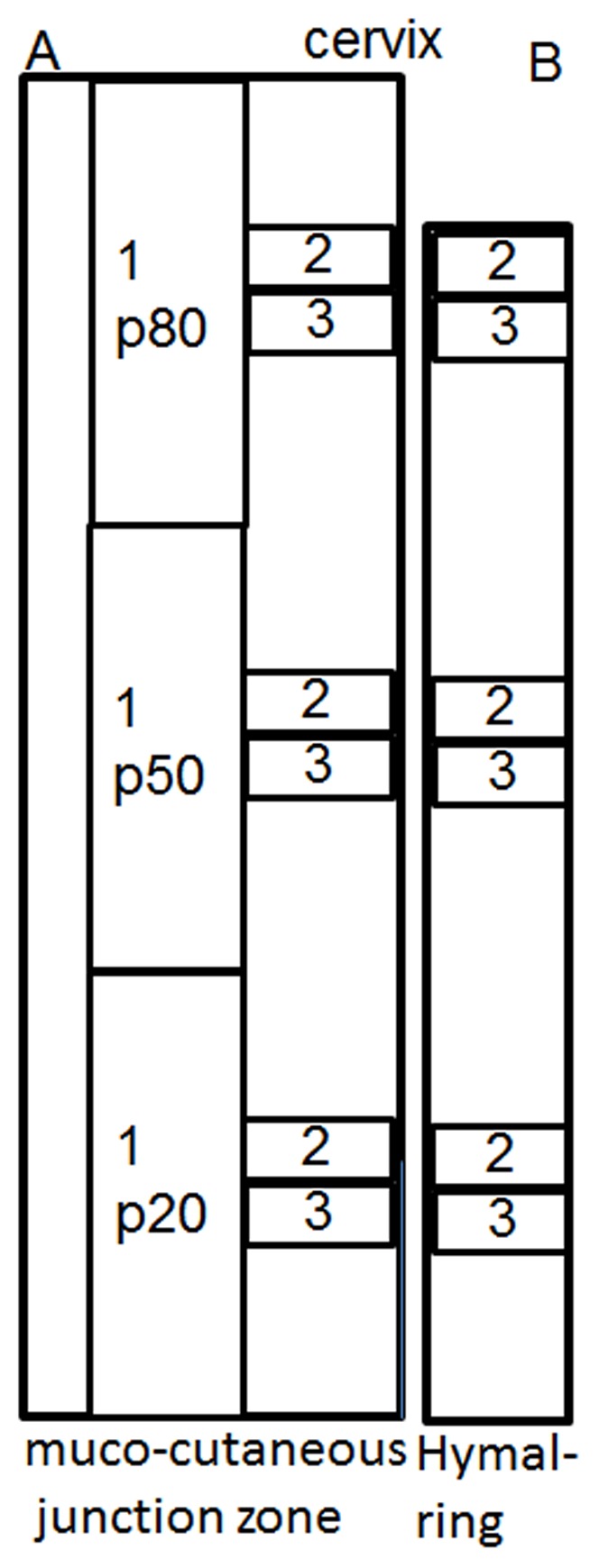
Schematic showing dissection of posterior vaginal wall. A. ovine. B. human. Specimen 1 was used for mechanical analysis, specimen 2 for biochemical analysis and, specimen 3 for histological analysis. Each was obtained at 20% (p20), 50% (p50) and 80% (p80) of total vaginal length. The total vaginal length in women was not excised, the tissue obtained closest to the cervix was deemed p80.

Vaginal tissue was harvested from 6 postmenopausal sheep that had delivered 3 lambs vaginally with the last lamb being delivered at least 12 months prior. A postmenopausal model was achieved by surgical removal of the ovaries. The animals were sedated with Medetomidine, anaesthesia was induced with Pentobarbitone sodium followed by isoflurane inhalation with ventilation (1.5–2.5%) in 100% O_2_. Antibiotics (Amoxicillin 1 g) were administered. The sheep were placed in dorsal recumbency and the wool shaved on the abdomen followed by skin prepping using Chlorhexidine, 70% alcohol and Betadine. A fentanyl patch was applied to secure pain relief. A 10 cm lower abdominal midline incision was performed and the ovaries removed. The abdominal fascia and subcutis were closed continuously, respectively, with 3/0 Vicryl followed by local anaesthetic (Bupivacaine, 5 ml) infiltration under the skin. 16 weeks after ovariectomy, animals were humanely euthanized by intravenous administration of Pentobarbitone sodium (150 mg/kg). A measure of maximum displacement of the vaginal wall was performed on the posterior vaginal wall 3 cm above the muco-cutaneous junction zone corresponding to point Bp of the pelvic organ prolapse quantitation (POP-Q) system and by traction on the cervix [10]. The POP-Q is an objective, site –specific system for semi-quantitative staging of pelvic support in women, used to allow standardisation of prolapse and is approved by the International Urogynecological Association (IUGA), the International Continence Society (ICS), the American Urogynecologic Society (AUGS) and the Society of Gynecologic Surgeons for the description of POP. The complete vaginal tract was removed from the 6 sheep immediately after euthanization; full vaginal thickness tissue was collected in a longitudinal manner from the posterior vaginal wall starting at the muco-cutaneous junction zone to the cervix. Tissue for biochemical, histological and biomechanical analysis was obtained at 20% (p20), 50% (p50) and 80% (p80) of the posterior vagina with p20 representing the distal third close to the hymen and p80 the proximal third close to the cervix (Fig. 1).

### HUMANS

Human tissue collection was approved by the Monash Health Human Research Ethics committee B. All women gave written informed consent. A thorough clinical history and pre-operative POP-Q parameters were obtained. Vaginal tissue was obtained from 7 women undergoing vaginal pelvic organ prolapse reconstructive surgery. Redundant vaginal tissue was excised from the midline and obtained at 20, 50, and 80% (at point of excision, Fig. 1) of the vagina in a similar manner to the sheep tissue acquisition for histology and biochemical analysis.

### HISTOLOGY

The explanted tissue was processed and stained with Masson's Trichrome to measure the percentage muscularis in each zone; p20, p50 and p80. Sections were viewed at X2 magnification and an area of muscularis was outlined in relation to the total area of the full vaginal wall thickness. Vaginal wall area was measured in µm^2^ from the epithelial to the adventitial margin using NIS-elements RA3.2 software.

The tissue was also stained with alpha smooth muscle actin (αSMA). Sections underwent dewaxing, rehydrating and antigen retrieval in citric acid buffer as described [11]. Endogenous peroxidase was quenched followed by Protein block (Dako, Denmark). The primary antibody (1∶400, Dako) was incubated for 1 hour, isotype control was applied at the same concentration (IgG2a, Dako). Secondary antibody (Mouse Envision Kit with HRP, Dako) was applied [12], colour was developed with 3,3′-Diaminobenzidine (DAB).

### BIOCHEMICAL ANALYSIS

Collagen content was measured by a hydroxyproline (Hyp) assay using 5×5 mm frozen tissue pieces. These were weighed, lyophilized for 4 hours, then digested with 1 ml papain (0.5 mg/ml in 0.1 M Na_2_HPO_4_, 5 mM EDTA, 5 mM Cysteine. HCl, pH 7.4) for 16 hours; supernatant was collected and total collagen content determined by hydrolysing in 6N HCl at 115°C for 4 hours followed by desiccation overnight [13]. After reaction with 0.05 mol/L chloramine-T (Sigma) and 10% (w/v in 2-methoxyethanol) ρ-dimethylaminobenzaldehyde (Sigma), Hyp was measured spectrophotometrically at 560 nm using a standard curve (L-Hyp standards (0–10 µg/mL) (Sigma)) and total collagen calculated using a Hyp to collagen ratio of 0.143∶1 [13].

The collagen type III/type I ratio was determined using a SDS-polyacrylamide gel electrophoresis (SDS-PAGE) using delayed reduction [14] as described [11]. Frozen tissue was thawed to room temperature, and 5×5 mm pieces were digested for 4 h in pepsin (Sigma) (0.5 mg/ml in 100 mM acetic acid, pH 2.5) at 4°C. Samples were electrophoresed for 1 h at 130 V. 5% v/v 2-mercaptoethanol (Sigma) was added to each well for 1 h; electrophoresis was then continued for 3 h at 130 V at 4°C. Gels were stained with Coomassie Blue R-250 solution, destained in 20% ethanol and 5% acetic acid. Images were taken using FujiFilm LAS-3000 software. The percentage of collagen III in each tissue was calculated from peak sizes using: Percentage type III collagen = Area α1(III)×1.12×100/[Area α1(III)×1.12]+ Area α1[I] [15]; the calibration factor 1.12 corrects for the colour yield from equal weights of the two collagen types [15].

The insoluble precipitate from the above papain-digested, centrifuged sample was used for indirect elastic tissue associated proteins (ETAP) analysis. This sample contains remaining insoluble collagen and ETAP (primarily elastin but also includes insoluble elastic-associated proteins fibulin, fibrillin and latent TGF binding protein) and was rinsed in PBS and distilled water. After freezing and lyophilization for 4 hours, the residual tissue was weighed (W_res_), and sent to Australian Proteome Analysis Facility for amino acid analysis. The weight of insoluble collagen in the residual tissue (W_res-col_) was calculated based on its corresponding Hyp amino acid amount. The percentage of ETAP in the tissue samples was calculated using: ETAP % = [(W_res_-W_res-col_)/W_dry_]×100.

Glycosaminoglycans (GAG) were measured by dimethylmethylene blue (DMMB) assay [16] using the same sample preparation as for collagen measurement. The GAG concentration in papain treated tissue digests was determined spectrophotometrically at 525 nm; Chondroitin sulfate C from shark cartilage (Sigma) was used as the standard (0–0.5 mg/mL).

### MECHANICAL TESTING

The tissue was stored at −20°C until testing, thawed overnight at 4°C and tested within 24 hours of defrosting. Freezing and thawing allows a more reliable assessment as specimens are tested under the same condition [17]. Vaginal tissue was dissected from sheep (n = 4–6) in the p20, p50, and p80 regions. Dogbone shaped samples (n = 1 replicate/region), central width and total length, 4 and 34 mm, respectively, were punched from the sheep tissue in the longitudinal axis and kept moist using PBS.

Sample thickness was measured at 3 positions using digital callipers for calculation of the initial cross sectional area. Uniaxial tensiometry was performed using an Instron Tensile Tester (5567Instron Corp, USA) and a 5 kN load cell. Samples were secured in pneumatic serrated jaws to prevent slippage and were set to a gauge length of 14 mm. Samples were preloaded at 10 mm/min to 100 mN and cyclically loaded from 0 to 1 N, 0 to 2 N, and 0 to 3 N, for 5 cycles each, at 20 mm/min and then extended to break [17]–[20]. Loading values were chosen to avoid damage to ovine tissue.

**Figure 2:**
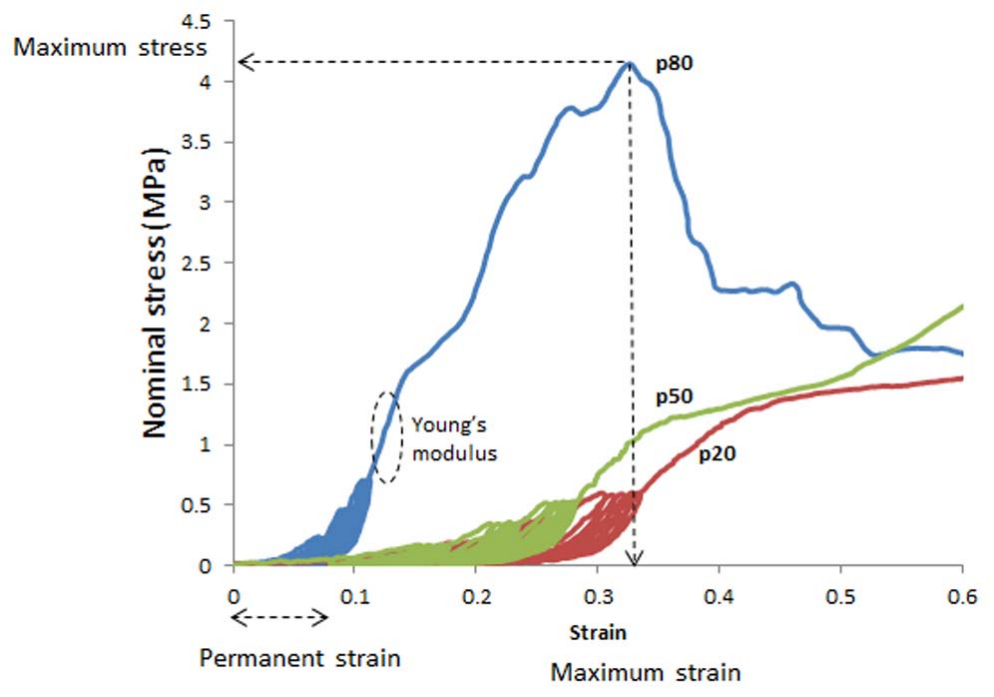
Typical nominal stress strain curves for a model of postmenopausal ovine tissue (ovariectomized parous ewes), indicating maximum stress and strain, Young's modulus, and permanent strain following cyclic loading for the p80 curve.

Stress-strain curves were plotted from the generated force and elongation data. Nominal stress (MPa, 1 Pa = 1 N/mm^2^) was calculated by dividing the force (N) by the initial cross sectional area (mm^2^) and strain by dividing the extension (mm) by the initial gauge length (mm). Young's modulus (MPa) was determined from the slope of the stress-strain curve in the linear region, immediately following cyclic loading and prior to yielding (Fig. 2). Permanent strain was calculated as the percentage increase in sample length following cyclic loading (Fig. 2). Maximum stress (MPa) was derived from the stress-strain curve and maximum strain (%) was calculated from the corresponding maximum strain, derived from the stress-strain curve (Fig. 2).

### STATISTICS

GraphPad Prism 6 was used for statistical analysis of the biochemical data, and R software (open source) for the biomechanical data. Results are reported as mean ±SEM for each experimental group. Two way ANOVA and post hoc test (Tukey's correction) were used for comparisons of biochemical and muscularis data between regions and between species, and for analysis of biomechanical data comparing sheep and region. Kolmogorov-Smirnov tests showed the normality required for each ANOVA was satisfied. P values<0.05 were considered statistically significant. Based on the total collagen as the primary outcome of interest with a power of 80% and an alpha level of 0.05, it was estimated that 6 subjects were necessary in each group (p20, p50, and p80) to detect a difference of 15% in the total amount.

## Results

All sheep were 4–5 years old and had delivered 3 lambs. The point corresponding to Bp could be moved to −1±0.1 cm, the cervix 1.5±0.3 cm indicating no significant prolapse in sheep.

The women's (n = 7) mean age was 71±8 years, median parity was 2 (1–4); mean time since menopause was 17±8 years. The human vaginal tissue showed stage 2 to 3 pelvic organ prolapse (Ba: 0.2±2.5, Bp: 0.0±1.8, C: −0.8±4.7).

**Figure 3:**
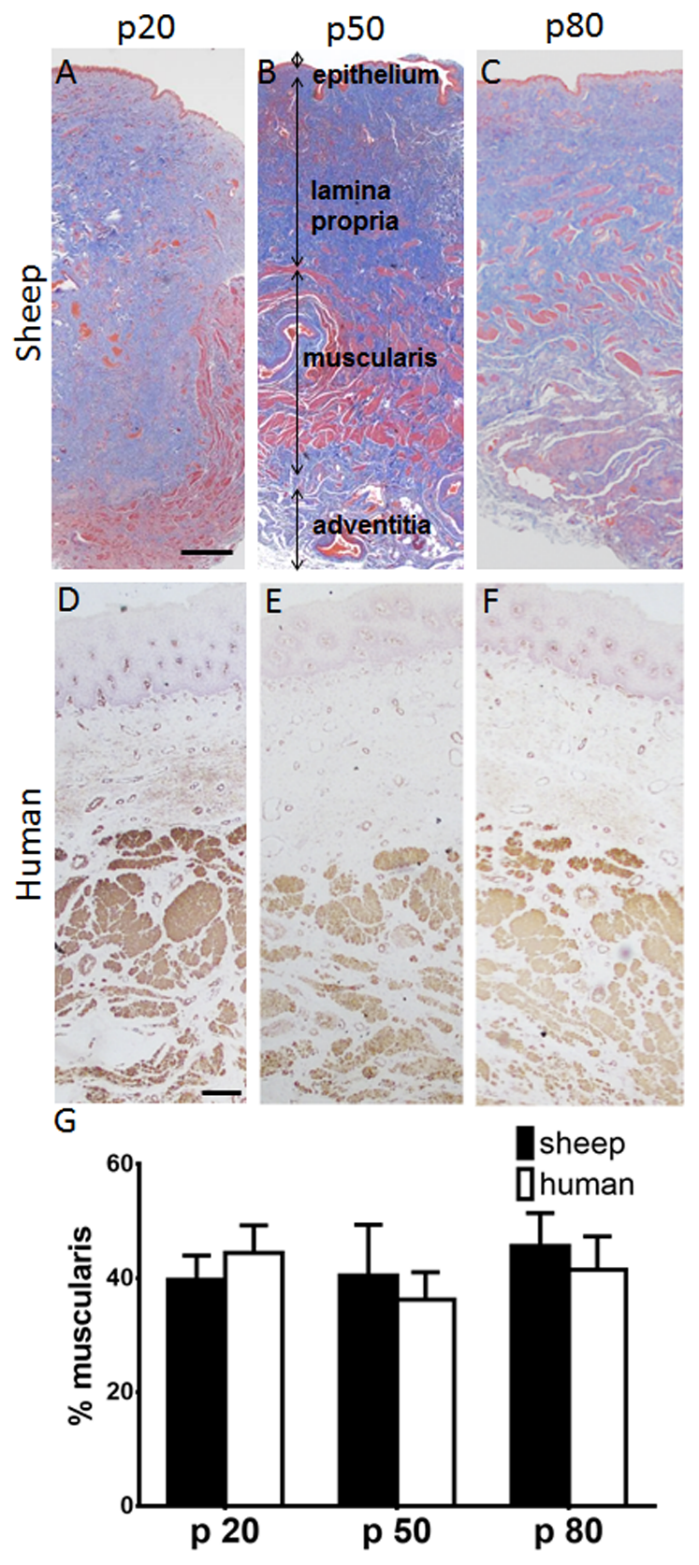
Masson staining of postmenopausal ovine vaginal wall at A. p20, B. p50, C. p80. αSMA staining of human vaginal wall D p20, E. p50, F. p80. % muscularis in G. ovine and human vaginal wall. Data are presented as mean (±SEM), n = 6/group each for sheep and for human. Scale bar is 250 µm.

Both ovine and human vaginal tissue showed the typical 4 vaginal wall zones of epithelium, lamina propria, muscularis and adventitia at the 3 regions examined (Fig. 3A–C). Masson's Trichrome (Fig. 3 A–C) and αSMC immuno-staining (Fig. 3 D–F) demonstrated the extent of muscularis in each full thickness vaginal tissue section for the 3 regions along the vaginal length (Fig. 1), with no significant difference in percent of muscularis between the anatomical regions from p20 to p80 in either human or ovine vaginal walls. In sheep, the percent muscularis varied from 39.7±4.2% at p20 to 45.6±5.8% (n = 6) at p80; in human samples, from 44.4±4.8% at p20 to 41.5±5.8% (n = 7) at p80 (Fig. 3G, H) with no significant difference between the two species for the 3 regions.

**Figure 4:**
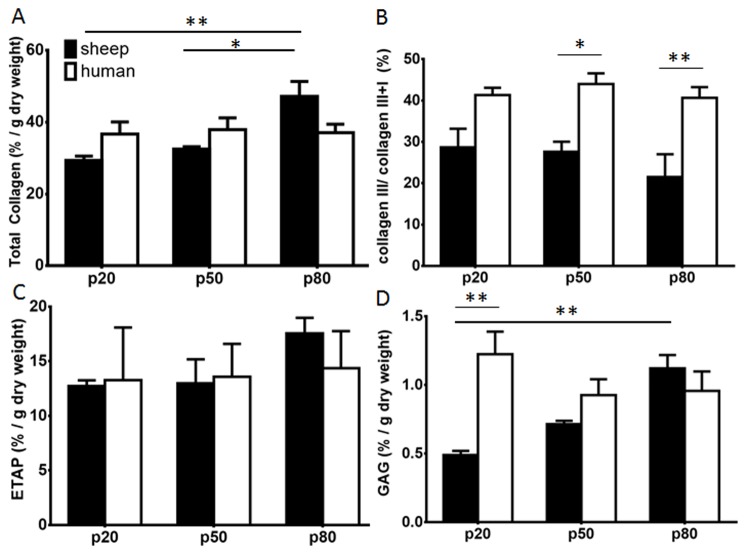
Biochemical analysis of the ECM content of postmenopausal ovine (black bars) and human (white bars) vaginal wall for the p20, p50 and p80 regions. A.% total collagen per dry weight assessed by hydroxyproline assay. B. % collagen III/(I+III) quantified by interrupted SDS-PAGE. C. % ETAP per dry weight by amino acid. D. % GAG per dry weight assessed by DMMB assay. Data are presented as mean (±SEM), n = 6/group each for sheep and human.* p<0.05, ** p<0.01.

The total collagen content was significantly higher (p<0.01) in the proximal region (p80) of ovine vagina compared to the distal (p20) and middle (p50) (p<0.05) regions (Fig. 4A). This difference was not observed in the human vagina; however the average total collagen content was comparable between the ovine and human regions of the vagina (Fig. 4A). Collagen type III, as measured by the collagen III/III+I percentage was 29±4.5% at p20, and 21±5.5% at p80 in the ovine vagina (Fig. 4B). There was no such trend in the human samples with all regions showing comparable levels of collagen type III, around 40% (Fig. 4B), which was significantly higher at p50 and p80 compared to ovine tissue (p<0.05, and p<0.01), respectively.

Similarly, no significant difference in total ETAP content was found along the ovine and human vagina (Fig. 4C). ETAP content was comparable between ovine and human tissue, approximating 15%.

In ovine vagina the GAG content was significantly higher (p<0.01) in the p80compared to the p20 region (Fig. 4D). In the human samples, there were no regional differences. In both ovine and human tissue samples, the % GAG content was not significantly different at p50 and p80 but at p20 GAG was significantly higher in human compared to sheep (p<0.01), but in both very low levels between 0.5 to 1.2% were observed.

**Figure 5:**
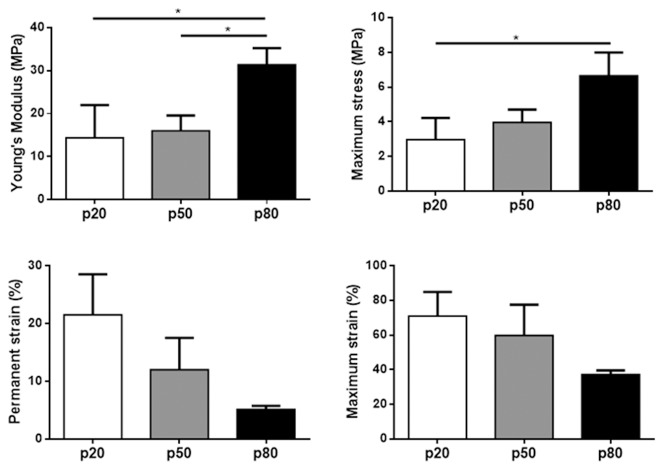
Biomechanical evaluation of vaginal tissues from ovariectomized (postmenopausal) parous sheep from the p20 (white bars), p50 (grey bars) and p80 (black bars) regions. A. Young's modulus (MPa). B. Maximum stress (MPa). C. Permanent strain (%). D. Maximum strain (%). Data is presented as mean (±SEM), n = 4–6/group. * p<0.05.

Due to small human sample sizes, biomechanical analysis was only possible for the ovine tissue. Young's modulus was highest in the p80 region indicating the proximal third of the vagina is the stiffest following cyclic loading (p<0.05) compared to p20 and p50 regions, which were of similar stiffness (Fig. 5A). Maximum stress (strength) was also highest in the proximal region, with significant differences between p20 and p80 regions (p<0.05) (Fig. 5B). Permanent strain, an indicator of tissue elasticity, did not show statistical differences along the vaginal length, however a trend of increasing elasticity was observed, with the proximal region being the most elastic (Fig. 5C). Maximum strain (extensibility) produced a similar non-significant trend, with the proximal region being the least extensible (Fig. 5D).

## Discussion

In this study we performed a detailed comparative analysis of the histological, biochemical and biomechanical properties (ovine only) along the postmenopausal ovine and human posterior vaginal walls. For the first time, we have directly compared the ECM composition with mechanical data.

We found significant differences between sheep and human vaginal tissues for collagen ratio and GAG, whereas there were no differences for total collagen and ETAP between the species at any of the vaginal regions. Several of the major ECM components were highest in the proximal region of the ovine vagina, particularly total collagen and GAG, although ETAP and collagen ratio did not differ significantly. In human tissue there were no significant differences along the vaginal wall for collagen, GAG or ETAP content. The p80 point in humans may sometimes have been 70–75% of the vaginal length, which may contribute to the lack of differences observed between the regions.

The vaginal wall consists of four layers, of which its major components have been quantified in several studies, however, it is difficult to compare existing studies given the range of techniques used; histology, immunohistochemistry and biomechanical analyses, and that the exact origin of the tissue is often not stated [21]. Many studies have relied on immunohistochemistry, which can only be regarded as semi-quantitative. In this study for the first time we undertook quantitative biochemical assays to accurately measure the major ECM proteins of vaginal tissue. We combined this quantitative biochemical analysis with histomorphometry to provide a comprehensive analysis of both ovine and human vagina.

Previous studies showed conflicting results in terms of human vaginal wall collagen content; some found no differences between women with or without POP [22], whereas others found a higher collagen content in women with POP [23]. Prolapse predominantly occurs in postmenopausal women and tissue analyses is often available for this reproductive status [21]. Our study has not included women without POP and neither has it compared with premenopausal controls, which are limitations. Collagen type I was the major ECM protein in a previous study [24] and in ovine and human vaginal tissue in this study, in contrast to others which showed collagen III as the dominant protein using immunoquantification [23]. Our quantitative biochemical analysis of ovine vaginal tissue indicated that total collagen was highest in the proximal region, which was associated with the highest maximum stress and Young's modulus, suggesting that the vaginal apex is the strongest and stiffest region, likely due to Collagen type I, known for conferring tissue strength [25].

Elastin is a major fibrillar protein of viscoelastic tissues [26]. ETAP, mainly elastin but also fibulin, fibrillins and latent TGF binding proteins, showed a trend towards higher values in the proximal ovine vagina, which was also the most elastic biomechanically (least permanent strain) compared to the distal regions. Higher elastin content was also found in women with POP compared with controls [27], whereas no differences were found in another study [22]. In this current paper, we have used a simple indirect gravimetric estimation of total elastic-like protein content that include predominantly elastin but also other elastin tissue-associated proteins (ETAP). All these elastic proteins are associated with elasticity and mechanical integrity of the tissue. Measurements were quantitated by subtracting the insoluble collagen content of the residual enzyme digested tissue pellets. There are more specific proteomic assays suggested for elastin determination including measurement of desmosine crosslinks, chromatographic methods, ninhydrin assays and possible ELISA assays that we are currently investigating. Nonetheless, the current method is relatively simple and we have previously shown it to be an accurate estimation of content that correlates well with mechanical attributes of the tissue [24].

The regional differences in biomechanical and biochemical properties observed along the length of the vagina could have developed through different forces experienced during previous deliveries. Our results in sheep are in line with a study in rats which showed significant regional differences of vaginal wall contractility [9]. We restricted our testing to the posterior vaginal wall tissue due to the different anatomical location of the urethral orifice between sheep and humans.

The sheep used in this study did not have significant prolapse in contrast to the women, however were comparable to the human study subjects in terms of parity and reproductive stage of life. Sheep have been recognized as a relatively suitable animal model for POP research [5], [6] due to similarities in the labour process, head- pelvis ratio and spontaneously develop POP.

The similarity in content for most components between sheep and women compliment earlier studies indicating that sheep can serve as a good model for vaginal surgery. Further studies are needed to show the dynamic behaviour of the different regions after i.e. vaginal surgery and whether the tissue composition varying along the vaginal length in sheep has an influence.
